# Enhancement of culled ewes’ meat quality: Effects of aging method and time

**DOI:** 10.1016/j.fochx.2024.101687

**Published:** 2024-07-23

**Authors:** Aristide Maggiolino, Lucrezia Forte, Vincenzo Landi, Mirian Pateiro, José Manuel Lorenzo, Pasquale De Palo

**Affiliations:** a Department of Veterinary Medicine, University of Bari Aldo Moro, Italy, Strada Provinciale per Casamassima km 3, 70010, Valenzano, Bari, Italy; b Centro Tecnológico de la Carne de Galicia, Rúa Galicia 4, Parque Tecnológico de Galicia, San Cibrán das Viñas, 32900 Ourense, Spain; c Area de Tecnoloxía dos Alimentos, Facultade de Ciencias, , Universidade de Vigo, 32004 Ourense, Spain

**Keywords:** Culled ewes, Meat quality improvement, Oxidative response, Tenderness, Flavour, Volatile compounds

## Abstract

This study assesses the impact of wet and dry aging, over 35 days, on various physico-chemical, colorimetric, oxidative, volatolomic, and sensory attributes of meat from culled ewes. Water holding capacity of dry-aged (DA) meat increased from day 28 and was significantly higher than wet-aged (WA) meat. Cooking loss of DA meat decreased, and it was lower than that of WA meat. Warner Bratzler shear force increased in DA meat but decreased in WA meat during aging. Higher oxidation product concentration in DA meat likely results from oxygen exposure. Some aldehydes and ketones peaked at day 7 in DA meat, surpassing levels in WA meat. Overall liking scores favored DA meat at day 14 and 21 but declined from day 14 to 35, coinciding with increased pentanal content. Dry aging could improve the acceptability of culled ewes' meat more than wet aging, but in short aging time (14 days).

## Highlights

• Culled ewes' meat has low market value.
• Dry and wet aging can improve some meat quality parameters.
• Dry aging improved tenderness and flavour of culled ewes' meat in the first 14 days.
• Dry-aged meat showed a higher degree of oxidation, compared to wet aging meat.
• Overall liking scores were higher in wet-aged meat at 28 and 35 days of aging.

## 1 Introduction

The peak of reproductive activity in ewes typically occurs around the age of 6 years, after which it gradually declines (Farrell et al., 2019). Consequently, at the conclusion of their productive phase, these animals are removed from the flock through culling. This culling process can account for a significant portion, up to 40%, of the entire flock (Chaturvedi et al., 2008). Meat derived from culled ewes often faces challenges in the market due to its relatively low price and limited appeal among consumers (Bhatt et al., 2012). One contributing factor to this may be its toughness, particularly since many animals are slaughtered with low body condition scores (Fruet et al., 2016). Traditionally, much of this meat is downgraded for use in pet food or rendering purposes. However, there has been notable progress in recent years towards redirecting a larger proportion of this product to higher-value markets for human consumption (Walsh, 2014). Meat quality tends to decrease with animal aging (Hopkins et al., 2006), with tenderness in particular worsening due to the relationship with collagen total amount and quality (Pannier et al., 2014). Additionally, the specific “ewe” flavour intensifies with age, along with the presence of off-flavors (Gravador et al., 2018). The flavour notes that characterize this meat are ‘mutton’ and ‘pastoral’ flavors. In particular, the first is given by short-chain and branched-chain fatty acids (BCFAs), whose content increases in older animals (Watkins et al., 2013). The ‘pastoral’ flavour is linked to pasture-feeding and is conferred by 3-methylindole (or skatole) (Sheath et al., 2022). Furthermore, there is a great variability in consumer preferences, linked to cultural and traditional habits that characterize each country (Prache, Schreurs, & Guillier, 2021). The market demand in Mediterranean countries is oriented towards light lambs, produced from dairy farms, and slaughtered at an early age, resulting in a pale meat with a delicate flavour, unlike the heavy lambs, slaughtered at an advanced age, fed on pasture and with a more intense flavour (Berge et al., 2003; Carrasco et al., 2009). Nevertheless, ewes meat is particularly consumed not only in Anglosphere countries and in Halal culture, but also in some Southern European countries, such as Italy, where it is used in some traditional dishes (Mandolesi et al., 2020). Moreover, ewes meat has been recognized as a nutritive food for human consumption, as a good source of protein and highly digestible essential amino acids, rich in micronutrients, but, above all, of essential fatty acids (Prache & Bauchart, 2015). In recent decades, the goal of increasing productivity has prompted ewes breeding systems to focus attention on few selected breeds that ensure higher production levels (Evenson & Gollin, 2003). These changes, in conjunction with the replacement of wool with synthetic fibres, endangered ewes and ovine sector to the point that many autochthonous ewe breeds of the Southern Italian regions are at risk of extinction (Sardaro & La Sala, 2021). Among merinized ewes' breeds, an iconic and well represented in Southern Italy is Gentile di Puglia. The survival of a breed depends on its ability to fulfil current and future market demand (Piedrafita et al., 2003). Therefore, increase the value of meat from autochthonous culled ewes could be a good strategy to decrease environmental impact in a circular economy perspective, to preserve biodiversity and, considering the costs related to culling, also to increase farmers' income. All the attributes involved in consumer acceptability of meat, ranging from the visual appearance to the sensory profile, can be modified by many factors, including aging (Beldarrain et al., 2020; Tateo et al., 2013). Aging can lead to an improvement in meat quality, in terms of tenderness but also of sensory profile, for the release of substances (peptides, free amino acids, free fatty acids), which act as a substrate for the synthesis of volatile aromatic compounds (Forte et al., 2024; Khan et al., 2015). The most employed methods of aging are the wet-aging (WA) and the dry-aging (DA). The dry-aged (DA) meat is perceived by consumers as a top-quality product and, since the process is more expensive, it is sold at higher prices than wet-aged (WA) meat. However, the process involves a sizeable exposure to oxygen and high moisture losses, that can be avoided by the vacuum packaging used in WA (Hastie et al., 2020; Hastie, Ashman, et al., 2021). As estabilished by Pavlidis et al. (2019), volatilomics represents a high-performance interpretation of volatile organic compounds present in foods, particularly in meat. Therefore, the aim of this study was to determine the effects of aging method (DA *vs.* WA) and time on culled ewes' meat quality, measured by physico-chemical, colorimetric, oxidative responses and volatolomic and sensory profiles.

## 2 Material and methods

### 2.1 Animal management, slaughtering and sampling procedures

All animals and procedures utilized in this research study were approved by the Ethical Committee for the Welfare of Animals used in scientific research at the Department of Veterinary Medicine, University of Bari (Approval n. 08/2021).

The trial involved 48 cull Gentile di Puglia ewes, slaughtered by the slaughter house staff at 7 ± 0.7 years of age (mean ± SD). The mean live weight was 82.5 ± 3.8 kg (mean ± SD). The slaughters took place in 2 different replicates, each six weeks apart (24 ewes per replicate). All animals were from the same farm and were culled by the farmer because they reached the maximum age set by farmer itself for ewes. Prior to slaughter, the ewes were fed for 60 days with *ad libitum* alfalfa hay (195 g/kg crude protein DM, 348 g/kg crude fiber DM) and a commercial pelleted concentrated mixture (barley, faba beans and corn; 21.3% crude protein, 11.4% crude fiber, 2.9% crude fat, and 6.9% ash; on a DM basis). Dry matter content (AOAC, 2005) (method 930.15) and ash content (AOAC, 2005) (method 942.05) were determined following standard protocols. Fat was evaluated according to the Soxhlet extraction procedure (AOAC, 2005) (method 991.36) and crude protein by Kjeldahl N × 6.25 procedure (AOAC, 2005) (method 968.06), as described by Maggiolino, Lorenzo, Quiñones, et al. (2019). All animals were transported and slaughtered at an abattoir approved by the European Community, situated 24 km from farm. This processes adhered to the European Community laws governing Animal Welfare in transport (1/2005EC) and the European Community regulation on Animal Welfare for the slaughter of commercial animals (1099/2009EC). Following slaughter, the hind limbs – from the coxofemoral joint to the distal part – were removed from each carcass and transported under refrigerate conditions (at a maximum temperature of 4 °C) to the laboratory and stored at 4 °C. After 24 h *post-mortem*, each hind limb was assigned in a balanced way to the two experimental groups: the 48 right hind limbs (24 per replicate) were subjected to DA and the 48 left ones (24 per replicate) to WA. The DA was carried out in static cell at 2 °C and 62% humidity. The WA was accomplished by vacuuming the limbs using a slightly permeable film, Besser Vacuum® film (Besser Vacuum, Dignano, Udine, Italy), characterized by a thickness of 65 μm, weight of 63 g/m^2^, and permeability equivalentl to 65 cm^3^/m^2^ per day under an oxygen atmosphere at a temperature of 23 °C and 85% relative humidity. For each aging method, each hind limb was randomly assigned to an aging time between those considered: 0, 7, 14, 21, 28 and 35 days (respectively D0, D7, D14, D21, D28 and D35). Hence, four limbs were assigned to each aging time in each aging method considered. At each aging time, meat samples were taken from DA and WA legs for the analysis. Samples for physico-chemical, colorimetric, oxidative profile and volatile compound analysis were taken from *rectus femoris* muscle. While samples for sensory analysis were collected from *vastus medialis* muscle. All the analyses were conducted on fresh meat.

### 2.2 Physico-chemical analysis

A portable pH meter equipped with a glass electrode shape (Carlo Erba pH 710; Carlo Erba Reagenti, Milano, Italy) was employed to easily penetrate tissues and measure pH levels. The pH meter underwent automatic calibration for muscle temperature using two buffer solutions with pH 4 and 7 before each measurement (Crison, Lainate, Italy). Cooking loss was assessed as described by De Palo et al. (2014). The centrifugation method was used to determine water holding capacity (WHC), according to De Palo et al. (2016). For Warner Bratzler shear force (WBSF) analysis, 3 parallelepipeds, each measuring 3 × 3 × 6 cm, were cut from each sample and cooked in a plastic bag in water bath at 80 °C until they atained an internal temperature of 75 °C. The internal temperature was measured using a copper constantin fine-wire thermocouple (Model 5SC-TT-T-30–36, Omega Engineering Inc.) fixed in the geometrical center of the sample. The cut samples were cylindrical, with a diameter of 1.27 cm, and the shear was parallel to the muscle fiber orientation. An Instron 1140 apparatus (Instron, High Wycombe, UK) provided with a computer was utilized, using a crosshead speed of 50 mm/min and a 50 N load cell. The mean value for each sample was derived from the three values obtained by shearing the three parallelepipeds. The results were expressed in kg.

### 2.3 Colorimetric analysis

Colorimetric variables were assessed based on the CIE L*, a*, b* (CIE, 1976) colour system using a Minolta CR-300 colorimeter (light source D65; Minolta Camera Co. Ltd., Osaka, Japan). The colorimeter was calibrated following the Hunter-Lab colour space system utilizing a white standard (L* = 99.2, a* = 1.0, b* = 1.9). Measurements were taken from a 0° viewing angle with A-pulsed xenon arc lamp with a reading surface of 8 mm diameter. Each measurement was performed on a freshly cut surface, folowing 1-h blooming period. Three measurements were taken from three distinct points for each sample, following the methodology described by De Palo et al. (2012). The measured colour parameters were used to determine changes in total colour (Δ*E*) and saturation difference.

(Δ_chroma_) (Boakye & Mittal, 1996), according to the following equations:

$$\Delta E={\left[{\left({L_{0}}^{*}-L^{*}\right)}^{2}+{\left({a_{0}}^{*}-a^{*}\right)}^{2}+{\left({b_{0}}^{*}-b^{*}\right)}^{2}\right]}^{1/2}$$

$$\Delta_{\text{chroma}}={\left({a_{0}}^{2^{*}}-{b_{0}}^{2^{*}}\right)}^{1/2}+{\left(a^{2^{*}}-b^{2^{*}}\right)}^{1/2}$$

### 2.4 Oxidative profile analysis

Lipid oxidation was assessed through the TBARS method (Buege & Aust, 1978) and expressed as milligrams of malondialdehyde (MDA) per kilogram of meat.

Two aliquots of homogenate (50 μl each), previously prepared for the TBARS determination, were added with 1 ml 10% trichloroacetic acid (TCA) and then centrifuged at 1200*g* for 3 min at 4 °C to measure protein oxidation, following the procedure described by Tateo et al. (2020). Protein concentration was calculated according to Biuret assay (Tokur & Korkmaz, 2007). The determination of hydroperoxides was carried out according the method described by Tateo et al. (2020). Results were expressed in micromoles per gram according to Buege and Aust (1978).

### 2.5 Volatile compounds analysis

At each aging time and for each aging method, meat samples weighing five grams were subjected to grilling at 150 °C for a duration of 5 min on each surface. An electrical griddle was used for the cooking process (model CG660; Delonghi, Treviso, Italy). To ensure proper cooking, the internal temperature of the sample needed to reach 70 °C, which was measured using a copper constantin fine-wire thermocouple (Model 5SCTT-T-30-36; Omega Engineering Inc., Norwalk, CT, USA) fixed in the geometrical centre of the sample following the procedure described by Maggiolino, Lorenzo, Marino, et al. (2019). Following the cooking and subsequent cooling, the samples were minced using a commercial grinder (Moulinex/Swan Holding Ltd., Birmingham, UK), weighed (1 ± 0.05 g) into 20 ml vials (Agilent Technologies, Santa Clara, CA, USA) with the addition of an internal standard (82 ng 2-octanol) and the vials were sealed with a laminated Teflon-rubber disc. The extraction of volatile organic compounds (VOC) was carried out using headspace SPME–GC–MS method as described by Natrella et al. (2020). The samples were then loaded into a Triplus RSH autosampler (ThermoFisher Scientific, Italy) for extraction at 37 °C for 15 min, using a divinylbenzene/carboxen/polydimethylsiloxane 50/30 mm SPME fiber assembly (Supelco, USA). Subsequently, the fibres were desorbed at 220 °C for 2 min in the injection port of the gas chromatograph (Thermo Fisher Scientific, Rodano, Italy), operating in splitless mode. The gas chromatograph was equipped with an ISQ Series 3.2 SP1 mass spectrometer (Thermo Fisher Scientific, Rodano, Italy). The compounds were separated on a VF-WAX MS capillary column (60 m, 0.25 mm i.d., 0.25 μm film thickness; Agilent, Santa Clara, CA, USA) under the following conditions: injection port temperature, 250 °C; oven temperatures, 35 °C for 5 min, then 1.5 °C/min to 45 °C, then 4 °C/min to 160 °C and a final increase up to 210 °C at 20 °C/min; the final temperature was held for 7 min. The mass detector was set at the following conditions: detector voltage, 1700 V; source temperature, 250 °C; ionisation energy, 70 eV; scan range, 40–300 amu. The tentative identification of the peaks was carried out using Xcalibur V2.0 software (Thermo Fisher Scientific, Rodano, Italy), Qual Browse in particular, by matching their spectra with the reference mass spectra from the NIST (National Institute of Standards and Technology, Gaithersburg, USA) library. The semi-quantitation of the compounds was carried out using the internal standard method and the amounts are expressed in μg/kg.

### 2.6 Sensory analysis

Sensory analysis was performed by an eight-person trained panel, who were selected based on their sensory acuity in accordance with the methods specified by the British Standards Institution (BSI, 1993) methods. The ethical committee approval for the sensory analysis was not necessary since it's not required for food products (Ministry of Health guidelines on studies conducted for the safety and properties of food products, 2018). The analysis was conducted in accordance with the Declaration of Helsinki, with appropriate protocols for protecting rights and privacy of all partecipants involved in the execution of the research. All subjects gave their informed consent *via* the statement: “I am aware that my responses are confidential, and I agree to partecipate in this survey” where an affermative reply was required to enter to the survey. They were able to withdraw from the survey at any time without giving a reason. At each experimental time, samples were collected from limbs and cooked as previously described for VOCs. Connective tissue and fat were removed, and the muscle cut into approximately 2 cm^3^ blocks. These blocks were then wrapped in pre-labelled foils and placed in a heated incubator until the tasting session. The tasting session was carried out following a specific order as outlined by MacFie et al. (1989) to account for carryover effects between samples. The panel test was organized in a single sitting sessions for each panellist, at each aging time. During each session, each panellist received four samples for each aging method, for a total of eight samples for each session. Each panellist tested all the samples for each aging method in duplicate, for a total of 16 tastings. The order of the samples was randomized by the sensory panel software, ensuring a different sequence for each panellist. The assessed samples were rated on a scale ranging from 1 to 10 for various sensory attributes, including tenderness (1 = extremely tough to 10 = extremely tender), juiciness (1 = extremely dry to 10 = extremely juicy), overall liking (1 = extremely disliking to 10 = extremely liking), sweetness, unpleasant taste, meaty odour and unpleasant odour, (1 = extremely weak to 10 = extremely strong).

### 2.7 Statistical analysis

The whole data set was tested for normal distribution and variance homogeneity (Shapiro-Wilk). The data set (excluding the sensory profile) was subjected to analysis of variance (ANOVA) using the General Linear Model (GLM) by SAS (2018) software (version 9.3, SAS Institute Inc., Cary, NC, USA), according to the following model:

$$y_{\mathrm{ijk}}=\mu +\alpha_{i}+A_{j}+T_{k}+{\left(A\times T\right)}_{\mathrm{jk}}+\varepsilon_{\text{ijkl}},$$

where y_ijk_ represents all parameters as dependent variables; μ is the mean; α_i_ is the single block random effect (1, …,4), A represents the effect of the j^th^ method of aging (j = 1, 2), T represents the effect of the k^th^ aging time (k = 1, …, 6) (for Δ*E* and Δ_chroma_ k = 1, …, 5), A × T represents the effect of the binary interaction between the two independent variables (jk = 1, …, 12) (for Δ*E* and Δ_chroma_ k = 1, …, 10) and ε_ijkl_ is the error. Subsequently, a Tukey test for repeated measures was carried out to evaluate the differences between the means during the aging time.

For the analysis of factors affecting each sensory trait, fixed terms for full models included aging and time. The experimental design factors of panellist, tasting round and session were fitted as random effects.

All means were expressed as square means and mean standard error. The significance level was set to *P* < 0.05.

## 3 Results

### 3.1 Physico-chemical and colorimetric variables

Results of physico-chemical and colorimetric responses are summarized in Table 1. The pH values did not reveal any difference between the two aging methods. In both aging methods pH dropped after D0 (*P* < 0.01). Only in DA meat a further pH decrease was recorded at D35 (*P* < 0.01). Aging time affected WHC only in DA meat, which showed higher values at D28 than at D0 (*P* < 0.01). At D28 and D35, DA meat had a higher WHC than the WA meat one. Cooking losses decreased from D0 to D35 in DA (*P* < 0.01). Differently, WA meat did not change this pattern during aging time, whereas had higher values than DA meat at D35 (*P* < 0.01). The WBSF decreased until D14 in DA meat, while increased at D21, when compared with D0 (*P* < 0.01). The WBSF of WA meat increased at D21, compared with D0 (*P* < 0.01). Moreover, WBSF values showed to be higher at D7 and D14 and lower from D21 to D35 in WA meat than the DA one (*P* < 0.01). Lightness and redness were not affected by aging time nor by aging method (*P* > 0.05). On the contrary, yellowness was affected by aging time in both aging methods, decreasing at D7 and D28 in DA meat, and only at D28 in WA (*P* < 0.01). In addition, yellowness was also affected by aging method, as WA meat showed higher values than DA meat from D7 to D35 (*P* < 0.01). The Δ*E* increased from D7 to D14 (*P* < 0.01) and at D28 and D35 was higher in DA meat than in WA meat (*P* < 0.05). The Δ_chroma_ increased in DA meat from D7 to D14 and further to D21, remaining constant in the subsequent time points (*P* < 0.01). Additionally, the Δ_chroma_ was higher in DA meat than in WA meat from D14 to D28 (*P* < 0.01).

**Table 1:** Effect of aging time, aging method, and their binary interaction on physico-chemical and colorimetric responses of culled ewes' *rectus femoris* (from the entire hind limb) aged for 35 days under dry and wet conditions (*n* = 8 samples for each aging time and for each aging method.

	Aging method	Aging time							Analysis of Variance		
		D0	D7	D14	D21	D28	D35	SEM^1^	A^2^	T^3^	A × T
*Physico-chemical responses*											
pH	DA	6.21 ^A^	5.64^B^	5.47^B^	5.42^B^	5.40^B^	5.25^C^	0.05	0.5889	<0.0001	<0.0001
	WA	6.22 ^A^	5.85^B^	5.56^B^	5.45^B^	5.40^B^	5.40^B^				
Water holding capacity (%)	DA	85.25 ^A^	88.25	89.35	90.15	92.45^B,X^	93.65^B,X^	4.33	0.0088	0.0024	0.0040
	WA	86.44	84.65	86.25	82.36	83.21^Y^	81.52^Y^				
Cooking loss (%)	DA	41.02 ^A^	45.35	46.23	39.21	36.14	33.28^B,X^	4.02	0.0092	0.0059	0.0071
	WA	40.25	42.15	41.32	40.65	41.36	40.66^Y^				
Warner Bratzler shear force (N)	DA	6.25 ^A^	5.15^B,X^	4.58^C,X^	6.35 ^A,X^	6.81 ^A,X^	6.58 ^A,X^	0.65	<0.0001	<0.0001	<0.0001
	WA	6.32 ^A^	6.44^Y^	6.15^Y^	5.45^B,Y^	5.22^B,Y^	5.62^B,Y^				
*Colorimetric responses*											
L*	DA	38.25	40.12	44.48	45.35	44.35	42.65	4.25	0.8422	0.0894	0.5223
	WA	37.26	41.25	45.23	45.33	44.65	44.36				
a*	DA	15.24	16.26	18.77	20.54	21.05	22.36	2.33	0.6256	0.1589	0.2283
	WA	16.45	17.22	16.98	17.01	16.55	16.89				
b*	DA	2.01 ^A^	0.45^B,X^	0.44^B,X^	0.40^B,X^	0.06^C,X^	−0.21^C,X^	1.03	<0.0001	<0.0001	<0.0001
	WA	2.23 ^A^	1.89^Y^	1.78^Y^	1.88^Y^	1.32^B,Y^	1.12^B,Y^				
Δ*E*	DA	–	3,49 ^A^	26,87^B^	40,55^B^	37,38^Bx^	37,49^Bx^	8.33	0.0241	<0.0001	0.0118
	WA	–	8,31 ^A^	32,00^B^	32,78^B^	27,73^By^	25,92^y^				
Δ_chroma_	DA	–	−14,15 ^A^	−58,10^BX^	−92,88^CX^	−103,40^CX^	−131,86^C^	15.64	<0.0001	<0.0001	<0.0001
	WA	–	−12,26 ^A^	−7,96^Y^	−8,65^Y^	−0,03^BY^	−5,48				

Different superscript letters in the same line show statistical differences (A, B, C: *P* < 0.01).

Different superscript letters in the same column, for each investigated pattern, show statistical differences (X, Y: *P* < 0.01).

1 SEM: standard error of mean; ^2^ A: aging; ^3^T: time; ^4^TBARS: thiobarbituric acid reactive substances.

### 3.2 Oxidation variables

**Fig. 1:**
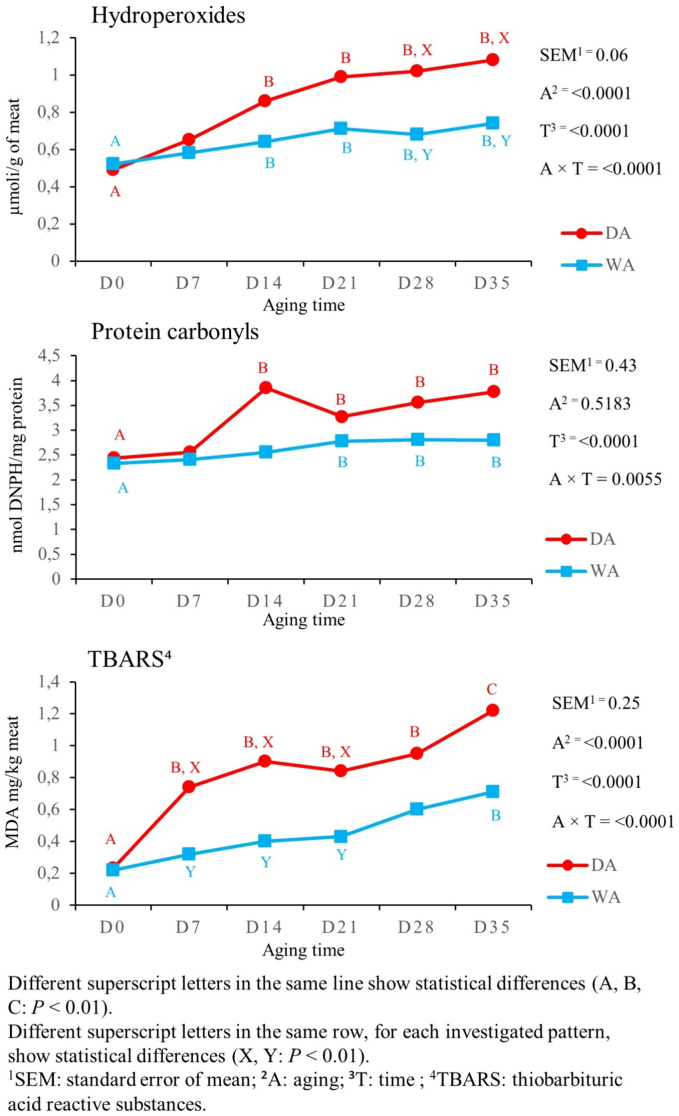
Effect of aging time, aging method, and their binary interaction on oxidative responses of culled ewes' *rectus femoris* (from the entire hind limb), aged for 35 days under dry and wet conditions (*n* = 8 samples for each aging time and for each aging method).

The results related to the oxidative profile patterns are shown in Fig. 1. In both aging methods hydroperoxides concentration increased at D14 than D0 (*P* < 0.01) and then remained constant up to D35 (*P* < 0.01). Higher hydroperoxides' concentration was detected in DA meat, compared with WA meat, at D28 and D35 (*P* < 0.01). Protein carbonyls recorded an increase at D14 in DA meat (*P* < 0.01) and at D21 in WA meat, when compared with D0 (*P* < 0.01). In DA meat, TBARS rose from D0 to D7 and reached the highest values at D35 (*P* < 0.01); whereas in WA meat TBARS' values slowly increased at D35, when compared with D0 (*P* < 0.01). The WA meat had lower TBARS' values from D7 until D21 than the DA one (*P* < 0.01).

### 3.3 Volatile organic compounds

**Fig. 2:**
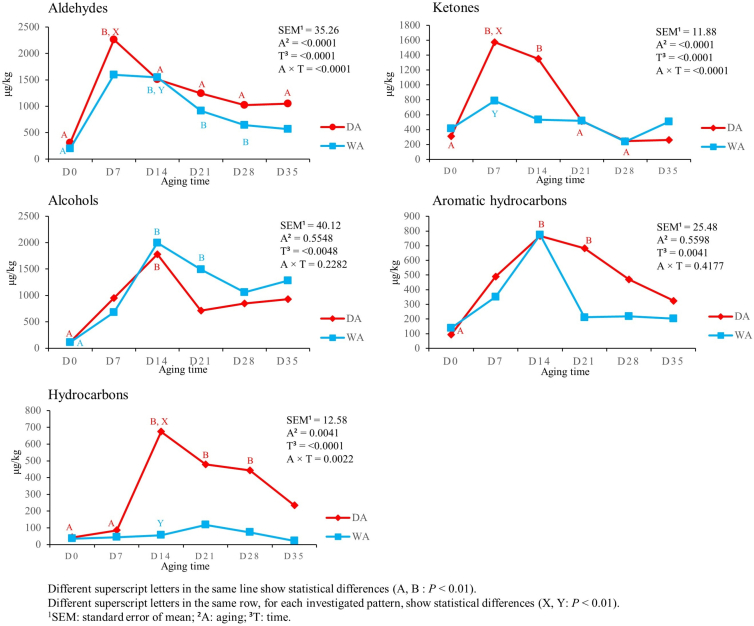
Effect of aging time, aging method, and their binary interaction on VOC families' content of culled ewes' *rectus femoris* (from the entire hind limb), aged for 35 days under dry and wet conditions, expressed in μg/kg (*n* = 8 samples for each aging time and for each aging method).

Fig. 2 shows the effect of aging time and aging method on VOC chemical families detected in DA and WA meat. The highest aldehydes production was reached at D7 in DA meat (*P* < 0.01) and at D14 in WA meat. In the following times, aldehydes' levels remained constant in WA meat (*P* < 0.01), while in DA meat exhibited a drop from D7 to D14 (*P* < 0.01) and remained constant afterwards. At D7, DA meat showed a higher content of aldehydes, when compared with WA meat (*P* < 0.01). Aging time affected ketones content only in DA meat, which reached the highest content at D7 and dropped from D7 to D14 (*P* < 0.01). At D7, DA meat had higher ketones content than WA meat (*P* < 0.01). While aging method did not show any difference in alcohols production, aging time affected it in both aging methods. Alcohols content displayed an increase from D0 to D14 in meat from both aging methods (*P* < 0.01). Aromatic hydrocarbons and non-aromatic hydrocarbons were affected by aging time only in DA meat, increasing from D0 to D14 (*P* < 0.01). Aging method affected only non-aromatic hydrocarbons at D14, when we detected higher levels in DA meat than in WA meat (*P* < 0.01).

**Fig. 3:**
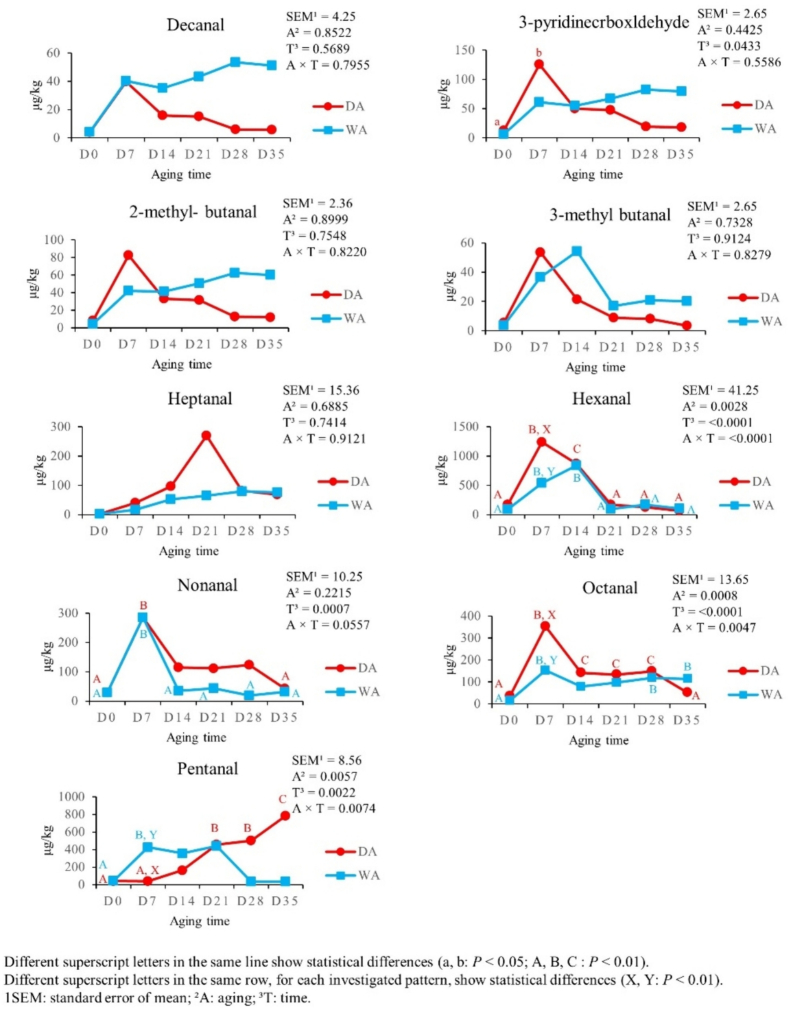
Effect of aging time, aging method, and their binary interaction on aldehydes content of culled ewes' rectus femoris (from the entire hind limb), aged for 35 days under dry and wet conditions, expressed in μg/kg (*n* = 8 samples for each aging time and for each aging method).

**Fig. 4:**
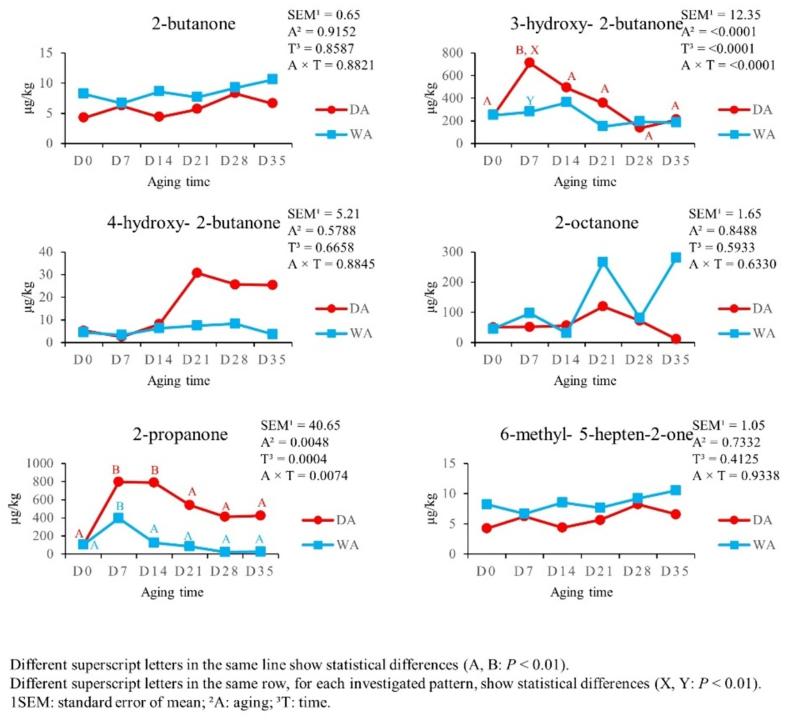
Effect of aging time, aging method, and their binary interaction on ketones content of culled ewes' rectus femoris (from the entire hind limb), aged for 35 days under dry and wet conditions, expressed in μg/kg (*n* = 8 samples for each aging time and for each aging method).

Fig. 3, Fig. 4 summarize the effects of the aging time and aging method on aldehydes (9 VOCs) and ketones (6 VOCs) content. Most aldehydes reached the highest levels from D0 to D7 in both aging methods (*P* < 0.01), except for pentanal in DA meat, which increased from D0 and D7 to D21 (*P* < 0.01), then at D21 (*P* < 0.01) and up to D35 (*P* < 0.01). From D14, aldehydes showed different trends. Hexanal decreased from D7 until D21 in both DA and WA meat (*P* < 0.01). In the following times of both aging methods, it remained stable (*P* < 0.01). Nonanal decreased only in WA meat from D7 to D14 (*P* < 0.01) and remained constant in the subsequent times (*P* < 0.01). Conversely, octanal decreased only in DA meat at D14 (*P* < 0.01) and up to D35 (*P* < 0.01). Hexanal and octanal content was higher in DA meat at D7, than the WA meat one (*P* < 0.01); while pentanal content was lower in DA meat at D7, when compared with the WA one (*P* < 0.01). So, several ketones were not affected by aging time (*P* > 0.05), except for 3-hydroxy- 2-butanone and 2-propanone. 3-hydroxy-2-butanone increased from D0 to D7 (*P* < 0.01) and dropped from D7 to D14 in DA meat (*P* < 0.01). Furthermore, 3-hydroxy-2-butanone levels were higher in DA meat at D7, than in WA one (*P* < 0.01). On the other hand, 2-propanone increased in DA meat from D0 to D7 (*P* < 0.01) and decreased from D7 to D14, up to D21 (*P* < 0.01). In WA meat, it remained constant (*P* > 0.05).

**Fig. 5:**
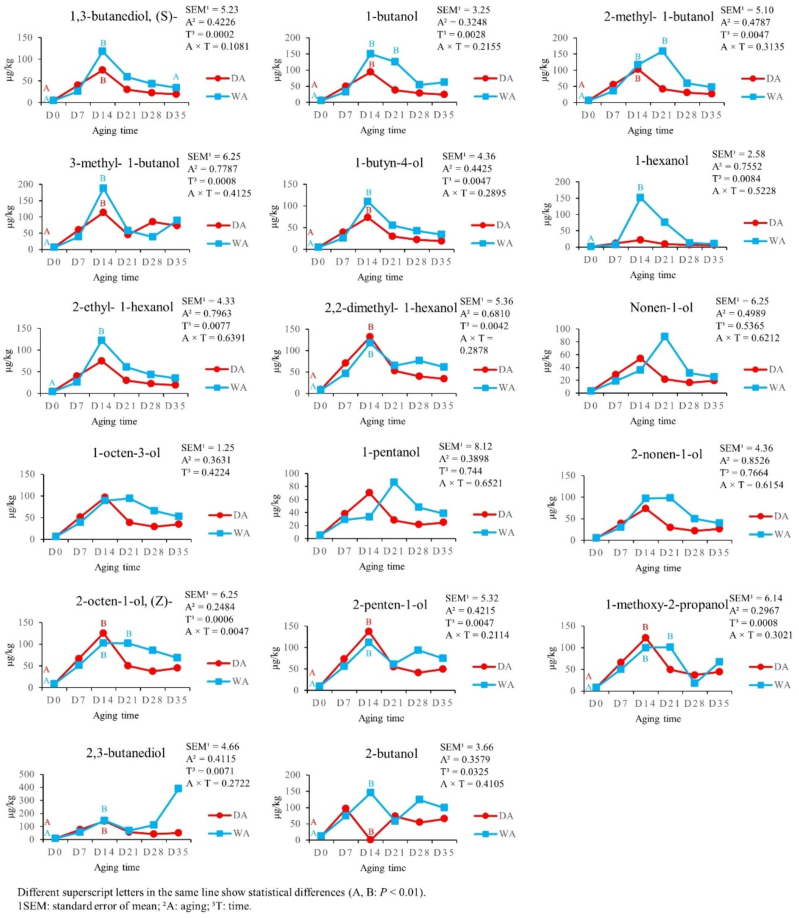
Effect of aging time, aging method, and their binary interaction on alcohols content of culled ewes' rectus femoris (from the entire hind limb), aged for 35 days under dry and wet conditions, expressed in μg/kg (*n* = 8 samples for each aging time and for each aging method).

In Fig. 5 is reported the effect of aging time and aging method on Alcohols (17 VOCs) content. 1,3-butanediol, (*S*)-, 1-butanol, 2-methyl-1-butanol, 3-methyl-1-butanol, 1-butyn-4-ol, 2,2-dimethyl-1-hexanol, 2-octen-1-ol, (*Z*)-, 2-penten-1-ol, 1-methoxy 2-propanol, 2,3-butanediol, and 2-butanol showed the same trend, reaching the highest contents from D0 to D14 in both aging methods (*P* < 0.01). The same significant increase was observed for 1-hexanol and 2,2-dimethyl-1-hexanol, but only in WA meat (*P* < 0.01). Nevertheless, aging method did not affect alcohols production (*P* > 0.05).

**Fig. 6:**
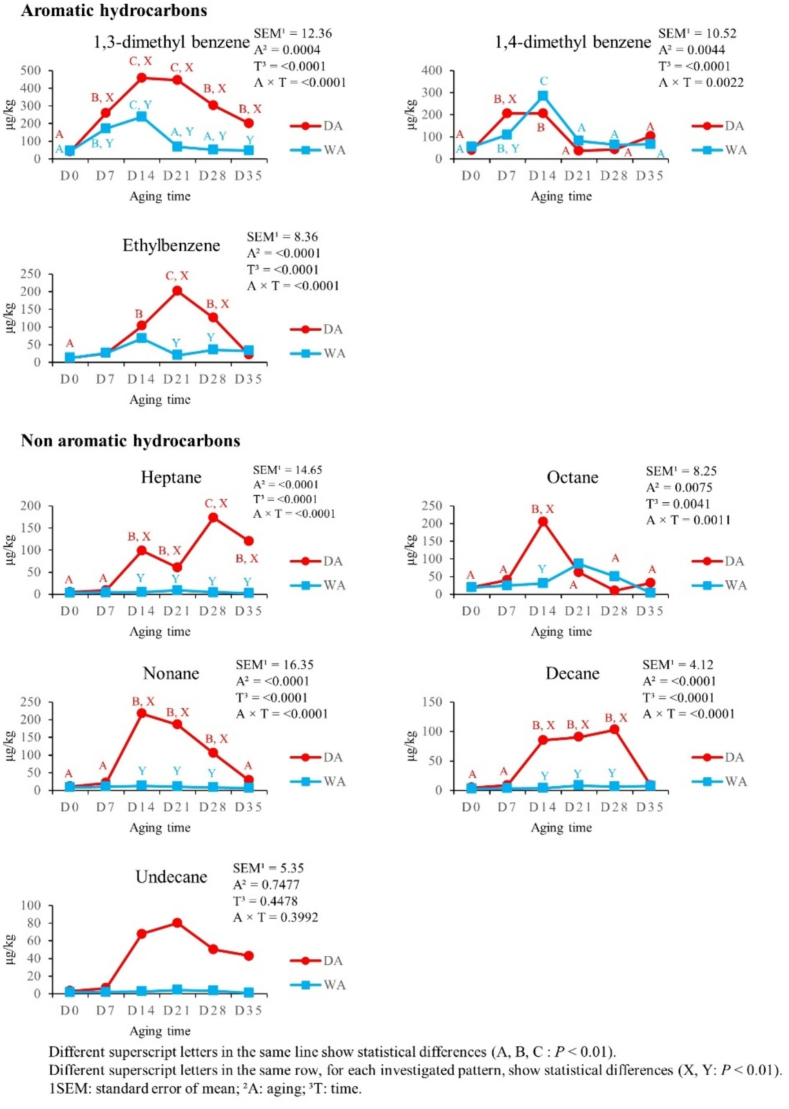
Effect of aging time, aging method, and their binary interaction on aromatic hydrocarbons and non aromatic hydrocarbons content of culled ewes' *rectus femoris* (from the entire hind limb), aged for 35 days under dry and wet conditions, expressed in μg/kg (*n* = 8 samples for each aging time and for each aging method).

Figs. 6 shows the effect of aging time and aging method on aromatic hydrocarbons (3 VOCs) and non-aromatic hydrocarbons (5 VOCs) content. 1–3-dimethyl-benzene increased from D0 until D14 in both aging methods (*P* < 0.01). In DA meat, its content decreased from D14 to D28 (*P* < 0.01), while in WA meat from D14 to D21 (*P* < 0.01). During the whole aging period, 1–3-dimethyl-benzene levels were higher in DA meat, than the WA meat ones (*P* < 0.01). 1,4-dimethyl-benzene increased from D0 to D7 in both aging methods (*P* < 0.01) and further only in WA meat from D7 to D14 (*P* < 0.01). A decrease in 1,4-dimethyl-benzene content was recorded from D14 to D21 in both aging methods (*P* < 0.01). Ethylbenzene was affected by aging time only in DA meat, increasing from D0 to D14 (*P* < 0.01), up to D21 (*P* < 0.01), and then decreasing at D28 (*P* < 0.01). Ethylbenzene content in DA meat at D21 and D28 was higher than the WA meat one (*P* < 0.01). Among non-aromatic hydrocarbons, heptane, octane, nonane and decane were affected by aging time only in DA meat, increasing from D0 and D7 to D14 (*P* < 0.01) and their levels at D14 were higher than those observed in WA meat (*P* < 0.01).

### 3.4 Sensory analysis

Results of sensory evaluation are reported in Table 2. In DA meat, juiciness decreased from D0 to D21 and even more at D28 (*P* < 0.01), while did not show modifications in the following times (*P* < 0.01). Conversely, in WA meat, juiciness did not show to be affected by aging time (*P* > 0.05). Moreover, DA meat was less juicy than WA meat from D21 to D35 (*P* < 0.01). Tenderness increased in DA meat from D0 to D7 (P < 0.01), decreased from D14 to D21 (*P* < 0.01) and then remained constant up to D35 (*P* < 0.01). In WA meat, tenderness received higher scores at D21, D28 and D35, when compared with D0 (*P* < 0.01). The DA meat showed higher tenderness than WA meat from D7 to D14 (*P* < 0.01). From D21 to D35, WA meat was more tender than the DA one (*P* < 0.01). Sweetness did not show to be affected by aging times nor by aging methods (*P* > 0.05). Meaty flavour perception in DA meat increased from D0 to D7 and even more at D14 (*P* < 0.01). From D14 to D28, meaty flavour perception in DA meat decreased (*P* < 0.01). Differently, in WA meat it increased from D0 to D21 (*P* < 0.01) and decreased from D21 to D35 (*P* < 0.01). Also in this case, DA meat showed higher meaty flavour than WA meat from D7 to D14 (*P* < 0.01). On the other hand, from D21 to D14, WA meat had higher meaty flavour than the DA one (*P* < 0.01). Unpleasant odours perception increased in DA meat from D0 to D21 (*P* < 0.01), and further grew up at D28 and D35 (*P* < 0.01). In WA meat unpleasant odours perception increased at D35, when compared with D0 (*P* < 0.01). The perception of unpleasant taste in DA meat increased from D0 to D21 and from D21 to D35 (*P* < 0.01); while in WA meat, aging time did not affect this pattern (*P* > 0.05). Both odour and unpleasant taste, were affected by aging method, showing higher scores in DA meat than in WA one from D21 to D35 (*P* < 0.01). Finally, overall liking was affected by aging time, increasing from D0 until D14 (*P* < 0.01) and decreasing from D14 until D35 (*P* < 0.01) in DA meat. Conversely, in WA meat overall liking increased from D0 and D7 to D14 (*P* < 0.01). Aging method affected also overall liking, which showed higher scores for DA meat at D14 and D21 (*P* < 0.01) and lower scores at D28 and D35 (*P* < 0.01), compared with WA meat.

**Table 2:** Effect of aging time, aging method, and their binary interaction on sensory evaluation of culled ewes' *vastus medialis* (from the entire hind limb), aged for 35 days under dry and wet conditions (*n* = 8 samples for each aging time and for each aging method).

	Aging method	Aging time							Analysis of Variance			
		D0	D7	D14	D21	D28	D35	SEM	A	T	A × T	
Juiciness	DA	7.12 ^A^	7.21	6.88	6.45^B, X^	5.44^C, X^	5.02^C, X^	0.65	0.0002	<0.0001	0.0004	
	WA	7.08	7.15	7.06	7.41^Y^	7.25^Y^	7.18^Y^					
Tenderness	DA	6.23 ^A^	7.12^B, X^	7.52^B, X^	5.58^C, X^	5.21^C, X^	5.02^C, X^	0.24	<0.0001	<0.0001	<0.0001	
	WA	6.18 ^A^	6.25 ^A, Y^	6.58^Y^	7.10^B, Y^	7.02^B, Y^	6.95^B, Y^					
Sweetness	DA	4.56	4.88	4.78	4.65	5.01	5.21	0.34	0.7225	0.6565	0.5221	
	WA	4.62	4.78	4.92	4.99	4.85	4.76					
Meaty flavour	DA	6.12 ^A^	7.52^B, X^	8.51^C, X^	6.52 ^A^	5.45^D, X^	5.61^D, X^	0.48	0.0041	<0.0001	<0.0001	
	WA	6.21 ^A^	6.54^Y^	6.25 ^A, Y^	7.12^B^	6.88^Y^	6.28 ^A, Y^					
Unpleasant odour	DA	3.12 ^A^	3.52 ^A^	3.47 ^A^	4.91^B, X^	5.82^C^	6.45^D, X^	0.81	0.0025	<0.0001	<0.0001	
	WA	3.51 ^A^	3.24 ^A^	3.45 ^A^	3.68 ^A, Y^	4.15	4.82^B, Y^					
Unpleasant taste	DA	3.24 ^A^	3.54 ^A^	3.52 ^A^	4.81^B, X^	5.54^B, X^	5.87^C, X^	0.22	<0.0001	<0.0001	<0.0001	
	WA	3.41	3.50	3.36	3.45^Y^	4.21^Y^	4.15^Y^					
Overall liking	DA	6.52 ^A^	7.25^B, X^	8.88^C, X^	7.12^B^	5.28^D, X^	4.06^E, X^	0.32	<0.0001	<0.0001	<0.0001	
	WA	6.48 ^A^	6.54 ^A, Y^	7.05^B, Y^	6.88	6.91^Y^	6.61^Y^					

Different superscript letters in the same line show statistical differences (A,B,C: *P* < 0.01).

Different superscript letters in the same column, for each investigated pattern, show statistical differences (X, Y: *P* < 0.01).

SEM: standard error of mean; ^2^ A: aging; ^3^T: time.

## 4 Discussion

There is a growing interest in enhancing culled ewes meat for human consumption (Fruet et al., 2016; Guerrero et al., 2018; Hastie, Ashman, et al., 2021; Hastie, Jacob, et al., 2021) and in preserving biodiversity by conserving autochthonous merinized ewes breeds (Ciliberti et al., 2021; Sardaro & La Sala, 2021). Since aging can improve various quality attributes of meat, inducing physical and chemical changes (Gómez et al., 2020), the quality of DA and WA meat from culled Gentile di Puglia ewes was evaluated.

Aging method and aging time both affected all the physico-chemical responses. The pH plays a key-role in qualitative characterization of aged meat (Hannula & Puolanne, 2004). Our results showed a pH decrease in both aging methods in the first week, while in the following time points no changes were recorded. However, during aging time, in both the methods, pH values never exceeded the threshold value of 5.8, above which a decline in ovine meat quality can be assumed (Johnson et al., 2005). It is already known that pH regulates the ability of meat to hold water (Cheng & Sun, 2008) and, according to our results, WHC and cooking loss were affected by the aging method. The WHC of DA meat increased at D28 and was higher than that of WA meat. On the contrary, cooking loss of DA meat decreased at D35 and was lower than that of WA meat. These results are consistent with the findings in DA and WA beef from Obuz et al. (2014) and Kim et al. (2020), who stated that the increase in WHC and the reduced cooking loss during DA could be due to the lower moisture content resulting from dehydratation and therefore lower water loss due to reduced availability during prolonged aging periods (Juárez et al., 2011). This is consistent also on the perception of juiciness assessed by panellists, that assigned lower scores in the last three weeks of aging, when compared with WA meat. Further evidence of this can be found in the appearance of legs already after the first week of DA, as dehydration was visually detectable. The high moisture losses involved in DA are primarily a problem in terms of yield, since it reduces the weight of the product and determines the formation of a dry crust on the surface, which is removed and discarded (Laster et al., 2008). To overcome the high moisture loss in DA, Hastie, Ashman, et al. (2021), Hastie, Jacob, et al. (2021)) suggested shorter aging period and the selection of ewes carcasses with higher fat cover and higher carcass weight. Moreover, high moisture losses have also implications on other quality attributes, such as tenderness (Cheng & Sun, 2008). The WBSF was affected by both aging method and time. At D7 and D14, WA meat had higher WBSF values than those of DA meat. From D21 the WBSF of DA meat increased, while that of WA meat decreased and the values of the latter were lower than those of the previous one. Sensory evaluation confirmed these results, inasmuch an improvement in tenderness was observed when WBSF values decreased in each aging method. Gürbüz et al. (2022) reported the same results in tenderness and WBSF of DA lamb loins. These authors observed an improvement in WBSF and tenderness after 7 days of DA, while no changes were recorded after 14 days. This suggested that, to obtain tender and juicy lamb meat, the extent of DA process could be reduced to 7 days, as also claimed by Feiner (2006) and this can also be applied to ewe meat. Regarding our results in WA meat, the plastic packaging used for vacuum could retain more moisture, resulting in an increased tenderness and juiciness as the aging period increases (Adegoke & Falade, 2005).

Tenderness and juiciness are strictly related to the recurrence of purchase by the consumers, whereas colour is usually important as driver for the first purchase and is often related to freshness and wholesomeness perception (Grunert et al., 2004). Myoglobin oxidation and surface drying reduce redness and lightness values, thus DA meat is usually darker and discoloured (Dikeman et al., 2013; Kim et al., 2016; Kim & Hunt, 2011). Although redness and lightness revealed no significant differences, Δ*E* and Δ_croma_ were notably affected by both aging time and method, showing an increase over time and especially during dry aging. The increase of these parameters during aging has been previously documented and is generally attributed to the increase in storage time and thus to the oxidation of myoglobin (Nerín et al., 2006; Zinoviadou et al., 2009). Regarding yellowness, it showed a decrease in both aging methods and higher values were observed in DA meat. Meat yellowness is mainly affected by lipid oxidation status (Maggiolino et al., 2021), so our recorded yellowness modifications are consistent with the oxidative profile of meat. The DA procedure involves meat exposure to oxygen and, consequently, triggers oxidative reactions. Moreover, fatty acid composition of ewes meat would also promotes oxidative processes (Kumar et al., 2015), as it consists of c.a. 45% of monounsaturated fatty acids (MUFA) and c.a. 10% of polyunsaturated fatty acids (PUFA) (Hajji et al., 2016). Therefore, in some aging point, DA meat had higher levels of hydroperoxides and TBARS, when compared with WA meat.

Oxidation-derived products, during the subsequent cooking process, influenced the synthesis of compounds, characterizing the typical flavour of cooked meat (Khan et al., 2015; Watanabe et al., 2015). A total of 40 VOCs were isolated and identified in cooked DA and WA ewe meat and were classified according to their chemical family as follows: 9 aldehydes, 6 ketones, 17 alcohols, 5 non-aromatic hydrocarbons and 3 aromatic hydrocarbons. The main source of VOCs is the lipid content, since most chemical groups – such as aldehydes, alcohols and ketones – resulted from lipid autoxidation processes and can themselves promote the formation of other compounds (Estevez et al., 2003). Therefore, the higher quantities of compounds that were overall found in DA, compared with WA, are related to the presence of oxygen, which activates oxidative processes. The predominant family in both aging methods were aldehydes, followed by ketones and alcohols. Aldehydes generally play a pivotal role in meat flavour and quantitatively overlap many other odour-active compounds in cooked meat (Mottram, 1998). The total amount of aldehydes was significantly affected by aging time, peaking at D7 and maintaining higher levels, than D0, up to D35 in DA meat and up to D14 in WA meat. Panea and Ripoll (2018) stated that there is a positive correlation between aldehydes and oxidation. This is consistent with our results, as aldehydes content was tightly related with TBARS trend in DA meat. The greater part of aldehydes have been frequently identified in the volatile profile of “ruminants meat” (Vasta et al., 2012), particularly hexanal and octanal (Vasta et al., 2013). Among aldehydes, in our results, hexanal represents the most abundant VOC generated in both aging methods. According to the current literature, hexanal is also the main VOC isolated from other species, such as foal, pork and beef (Dominguez et al., 2014; Ma et al., 2012; Maggiolino, Lorenzo, Marino, et al., 2019; Ramírez et al., 2004). In our study, hexanal, nonanal and octanal showed the same behaviour, peaking at D7 in both aging methods. Therefore, hexanal's trend probably affected the significant differences of the whole aldehyde's family. These findings are inconsistent with the results from the study conducted by Li et al. (2021). The authors reported that with the extension of the aging period of beef loins, heptanal, octanal and nonanal content increased in DA meat and decreased in WA meat. It is important to consider that volatile compounds and flavour development is also affected by fatty acids composition of meat (Van Ba et al., 2012). Hexanal and pentanal, which were commonly identified in lamb meat, may derive from the degradation of C18:2*n*–6 fatty acid (linoleic acid) (Elmore et al., 2005); while octanal and nonanal probably originate from the oxidation of C18:1*n*–9 (oleic acid) (de Santa Olalla et al., 2009). Hexanal, octanal and nonanal play a pivotal role in sensory profile, since are usually associated with odours described as meaty, green, fatty and sweet (Frank et al., 2016). Differently, pentanal plays a negative role in sensory assessment and it could be responsible of rancid off-flavour (metallic, green, earthy, beany) (Stetzer et al., 2008). While in WA meat, pentanal followed the trend described for the family; in DA meat it showed a different behaviour, increasing at D21 and even more at D35, reaching the highest quantity. These results indicate that as the by-products of lipid oxidation accumulate, mostly in DA meat, pentanal content increase and could result in a sensory quality impair (Stetzer et al., 2008). Aldehydes have a low odour detection threshold; therefore, they can strongly affect meat aroma and sensory evaluation (Van Ba et al., 2012), so the outcomes of volatile compounds analysis were supported by those of panel test. Meaty flavour was most perceived in the first two weeks of DA, with the highest hexanal content. From D21, a worsening of meat sensory profile was noticed, due to unpleasant odours and flavors perception, probably linked to the simultaneous accumulation of pentanal. Differently, WA meat received the highest score for meaty flavour at D21. Although meaty flavour in all times received lower scores than those of DA, unpleasant odour and taste were almost not perceived.

The second most abundant chemical group detected in samples were ketones. Total ketones increased in DA meat at D7, reaching higher quantities compared with WA meat, decreased at D14 and remained constant in subsequent times. The main reactions involved in the production of these VOCs are fatty acids oxidation, Strecker's degradation of amino acids, but also lipolytic activity and alkane degradation by microbial metabolism (β-oxidation) (Casaburi et al., 2015; Sirtori et al., 2020; Weenen, 1998). Moreover, it is known that the production of ketones is positively related with intramuscular fat content. Regarding the impact on meat flavour, ketones could be responsible of sweet, spicy, buttery, cheese, caramel and fatty notes (Ba et al., 2014; Gorraiz et al., 2002; Maggiolino, Lorenzo, Marino, et al., 2019). The most represented compounds among ketones were 3-hydroxy-2-butanone, and 2-propanone. These two compounds were also detected in DA and WA beef loin (Li et al., 2021). Consistently with the results of Li et al. (2021), in our study the concentration of 3-hydroxy-2-butanone was significantly higher in DA, than that in WA. The 3-hydroxy- 2-butanone gives a good buttery odour in beef (Machiels et al., 2004). On the other hand, while Li et al. (2021) reported a significant increase of 2-propanone in both aging methods, in our results this compound followed a different trend. In DA meat, 2-propanone increased at D7 and decreased at D28; while in WA meat it remained constant. The synthesis pathways of aldehydes and ketones are the same and these reactions occurs mainly during cooking but could also start during aging (Resconi et al., 2012).

Alcohols showed the highest number of compounds. Most of them followed the same trend in both aging methods, showing a significant increase from D14. The source of these compounds primarily stems from the oxidation of lipids, particularly the degradation of oleic and linoleic acid (Feng et al., 2020). However, certain alcohols, such as 1-methoxy-2-propanol, are typically generated from branched-chain amino acids and pyruvate catabolism (Estevez et al., 2003). Considering their high odour threshold limit, the contribution of alcohols to overall flavour is lower than other compounds (Domínguez et al., 2014). Some of these compounds, such as 1-hexanol, 1-octen-3-ol and 1-pentanol has been also identified in lamb, donkey and foal aged meats (Insausti et al., 2021; Maggiolino et al., 2020; Tateo et al., 2020). The 1-octen-3-ol, responsible of mushroom aroma, is considered one of the most characteristic volatile compounds in lamb meat, while in our results – although present – it showed to be not affected by aging time and method. The 1-hexanol contributes to herbal and fatty notes perception (Karabagias, 2018) and showed a significant increase only in WA meat.

The total content of aromatic hydrocarbons in DA showed a significant increase at D14 and until D21 remained constant, while no differences were detected during WA time. Also in this case, the significant differences between aging method, are probably due to the reduced lipid oxidation in WA. Considering the single compounds, in both aging methods 1,3-dimethyl-benzene and 1,4-dimethyl-benzene increased at D7. In DA meat, only ethylbenzene increased at D14. Whereas aromatic hydrocarbons can provide an important contribution to meat flavour, due to their low odour threshold (Karabagias, 2018), non-aromatic hydrocarbons are characterized by a high odour threshold, therefore their contribution to ultimate flavour could be considered irrelevant. The total non-aromatic hydrocarbons content significantly increased at D14 of DA and remained constant in the subsequent time points. Within this class, the most abundant compound was nonane. Except for heptane, which peaked at D28, octane, nonane and decane peaked at D14, decreasing thereafter. Scores attributed for overall liking could assert the idea that DA can improve the acceptability of culled ewe meat more than WA, but with shorter aging periods. In fact, the extension of the DA period beyond two weeks, leaded to a progressive decay of overall liking scores.

## 5 Conclusions

The outcomes of the present study revealed that dry aging in the initial 14 days led to an enhancement in terms of tenderness and flavour. Conversely, an extended period of dry aging adversely affected juiciness and tenderness, and, as a consequence of increased oxidation, led to the development of compounds responsible for unpleasant odours and flavors. Although vacuum packaging in the WA meat moderated oxidative reactions and dehydration even during longer aging periods, the improvement in quality attributes was milder compared to that observed in the DA meat. These findings suggest that both wet and dry aging have the potential to enhance and add value to a product that is currently undervalued in the market. Further research is warranted to evaluate additional quality parameters, such as fatty acid profile, or the effect of other yet unexplored aging methods.

## Ethics approval

All animals and procedures employed in this study received approval from the Ethical Committee for Welfare of Animals employed in scientific research of the Department of Veterinary Medicine of the University of Bari (Approval n. 08/2021).

Sensory evaluation was conducted with appropriate protocols for protecting rights and privacy of all partecipants involved in the execution of the research (no coercion to partecipate, full disclosure of study requirements and risks, written consent of partecipants, no release of partecipant data without their knowledge, ability to withdraw from the study at any time).

## Data and model availability statement

None of the data were deposited in an official repository**.**

## Financial support statement

This study was carried out within the Agritech National Research Center and received funding from the European Union Next-GenerationEU (PIANO NAZIONALE DI RIPRESA E RESILIENZA (PNRR)—MISSIONE 4 COMPONENTE 2, INVESTIMENTO 1.4—D.D. 1032 17/06/2022, CN00000022). This manuscript reflects only the authors' views and opinions, neither the European Union nor the European Commission can be considered responsible for them.

## CRediT authorship contribution statement

**Aristide Maggiolino:** Writing – review & editing, Writing – original draft, Visualization, Investigation, Conceptualization. **Lucrezia Forte:** Writing – original draft, Software, Methodology, Investigation, Data curation. **Vincenzo Landi:** Supervision, Methodology. **Mirian Pateiro:** Writing – review & editing. **José Manuel Lorenzo:** Writing – review & editing. **Pasquale De Palo:** Writing – review & editing, Supervision, Project administration.

## Declaration of competing interest

The authors declare that they have no known competing financial interests or personal relationships that could have appeared to influence the work reported in this paper.

## Data Availability

Data will be made available on request.
